# Subclinical Tuberculosis Diagnosis in Lusaka, Zambia: A Case Report

**DOI:** 10.7759/cureus.96269

**Published:** 2025-11-06

**Authors:** Nsala Sanjase, Brian Shuma, Evelyn K Ngandu, Seke Muzazu, Kondwelani Mateyo, Monde Muyoyeta

**Affiliations:** 1 Tuberculosis, Centre for Infectious Disease Research in Zambia, Lusaka, ZMB; 2 Epidemiology and Public Health, Centre for Infectious Disease Research in Zambia, Lusaka, ZMB; 3 Internal Medicine, University Teaching Hospital of Zambia, Lusaka, ZMB

**Keywords:** clinical presentation, computer-aided diagnosis, contact investigation, diagnosis, patient perspectives, subclinical tuberculosis, transmission

## Abstract

Identifying subclinical tuberculosis (TB) remains a significant challenge due to the absence of typical symptoms, the lack of diagnostic tests to distinguish disease states along the TB clinical spectrum, and the inability of plain radiography to pick up subtle changes associated with it. In this paper, we present two instances of subclinical TB identified in a high TB burden setting, highlighting the difficulties in diagnosis, challenges in treatment adherence and completion, and the risk of transmission.

## Introduction

Mathematical modelling has suggested that 68% of the global tuberculosis (TB) burden is subclinical, a preclinical asymptomatic TB state in which patients do not present with any signs and symptoms of TB but may have radiological evidence of disease and evidence of metabolically active mycobacterial TB, i.e., a positive Xpert result or TB culture result [1]. This disease phase particularly presents a unique challenge in TB epidemiology due to the limited knowledge about its presentation, diagnosis, and transmission dynamics [2]. Subclinical TB is a reservoir of future active TB cases, a disease state in which patients present with signs and symptoms of TB, such as fever, weight loss, night sweats, and cough, and have either radiological evidence of disease or microbiological evidence of disease (Xpert positive or culture positive), as a proportion of individuals may eventually progress to active disease, especially in the presence of immune compromise [3]. In addition, studies have shown evidence of TB transmission from asymptomatic subclinical TB patients, and this has the potential to contribute to continued TB transmission and progression to active disease [2,4]. However, it is worth noting that epidemiological data on subclinical TB prevalence vary across regions and are influenced by factors such as population density, TB incidence, and healthcare access [1].

Accurate diagnosis of subclinical TB remains a challenge. This is because individuals with subclinical TB do not present with classical signs and symptoms or may have unrecognizable symptoms [5]. To make this diagnosis, one must demonstrate the presence of viable *Mycobacterium* through a positive TB culture, a positive mycobacterium smear, or GeneXpert result [6], with or without chest radiographic changes. However, these tests are only routinely done as a diagnostic workup for patients with classical symptoms of TB [7]. Chest radiography may equally be useful, especially when coupled with computer-aided diagnosis (CAD), which is able to identify subtle changes [8]. Subclinical TB also presents a substantial challenge to global TB control efforts due to its potential to fuel future TB epidemics [1,4]. Improved understanding of its epidemiology, development of accurate diagnostic tools, and targeted management of high-risk individuals are essential for effective TB control and elimination. Addressing subclinical TB not only reduces the individual burden of disease but also contributes to breaking the cycle of TB transmission, thereby advancing global efforts toward ending the TB pandemic.

The novelty of this case report is that it presents two subclinical TB cases, a disease state that is underrecognized yet has important implications for diagnosis, treatment, transmission dynamics, and public health.

## Case presentation

Case 1

A 28-year-old black African male presented without symptoms suggestive of TB, i.e., cough, fever, night sweats, and weight loss. He reported no history of close or household TB contact.

He was HIV-negative and had no history of TB, diabetes, hypertension, asthma, or any other chronic medical conditions. However, he was on pre-exposure prophylaxis for HIV infection prevention (tenofovir 300 mg and lamivudine 150 mg) with poor adherence. He admitted to a history of drug abuse, alcohol abuse, and cigarette smoking. He was married with two children under the age of five and lived in a one-room house with inadequate ventilation, in a densely populated area of the capital city, Lusaka. He worked as a garbage collector.

**Figure 1:**
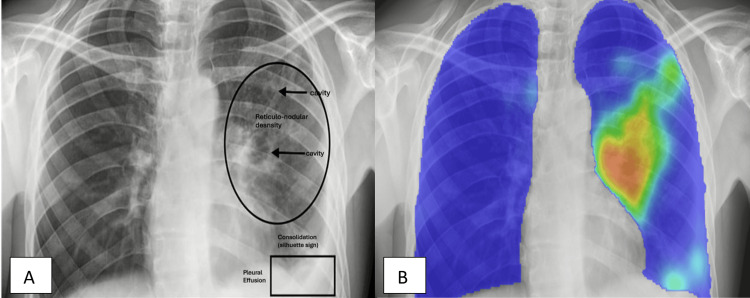
Case 1 chest X-ray: pulmonologist vs. CAD interpretation (A) Pulmonologist chest X-ray interpretation showing cavitation and reticulo-nodular infiltrates (suggesting an infection) and a silhouette sign on the left hemidiaphragm (suggesting a left lower lobe consolidation). (B) Chest X-ray with the CAD heatmap showing areas of possible abnormality highlighted in green and orange and areas with normal lung parenchyma in blue.

On physical examination, he had normal vital signs. Of note was a greyish brown discoloration of his complexion, which was reportedly a change from his previous brown complexion. In addition, he had reduced breath sounds on the right side of the chest on auscultation. These findings prompted chest X-ray examination, which showed cavitations, reticulo-nodular infiltrates, and a silhouette sign on the left hemidiaphragm as interpreted by a pulmonologist blinded to the CAD reading. The CAD heatmap was in alignment with the pulmonologist‘s interpretation (Figure 1); however, we did not have the heatmap evaluated by a radiologist to further validate it.

The interferon-gamma release assay (IGRA) was negative, as was the confirmatory rapid HIV test. Sputum GeneXpert Ultra revealed the presence of *Mtb* (trace) with indeterminate rifampicin resistance, while the sputum smear was scanty. Treatment was commenced with a fixed-dose combination of anti-tuberculous treatment for drug-sensitive TB containing rifampicin 600 mg, pyrazinamide 1600 mg, ethambutol 1100 mg, and isoniazid 300 mg, per day based on weight at the time of diagnosis. However, he defaulted one month after the initiation of treatment and was lost to follow-up. On contact investigation, his two children were clinically diagnosed with TB, both having cough, poor appetite, listlessness, malnutrition, and chest X-ray changes suggestive of TB. They were both started on treatment, reported clinical resolution of symptoms, and have since completed treatment.

Case 2

A 27-year-old black African female volunteered to participate in a healthy cohort study. At baseline, she presented with no history of TB symptoms or close or household TB contact.

The patient was HIV-negative and had no history of diabetes, hypertension, asthma, or any other comorbidities. She, however, reported a history of taking pre-exposure prophylaxis for HIV infection prevention (tenofovir 300 mg and lamivudine 150 mg). On review of past medical records, the patient had been lost to follow-up in a previous study where her sputum had been identified as culture-positive for TB. She had presented with a 12-day history of cough and fever, which was treated as a lower respiratory tract infection and resolved with antibiotics. She remained asymptomatic thereafter for over a year. Efforts to contact her had been futile, and she had not commenced on anti-tuberculous treatment.

The patient reported a history of alcohol misuse with no associated smoking or other recreational drug use. The patient lived in a two-bedroom, well-ventilated house with two other occupants in a TB-endemic township. At the time of the subclinical diagnosis, she worked as a community health worker at the local hospital, attending to healthy mothers seeking antenatal care services.

On physical examination, she was healthy-looking and was not in respiratory distress. A review of all systems did not indicate any abnormalities. Sputum examination was positive for *Mtb* on GeneXpert (very low), while the sputum smear was scanty positive. While refusal rates in our settings are generally low, the patient was not agreeable to the diagnosis of TB, as this was a rare diagnosis of TB in the absence of symptoms, and opted to seek a second opinion at a private health facility. She reported that the sputum sample examined at the private hospital was equally Xpert positive. The patient underwent extensive counselling through the standard of care and a daily observed treatment plan where a study staff ensured that the patient took her medication daily.

**Figure 2:**
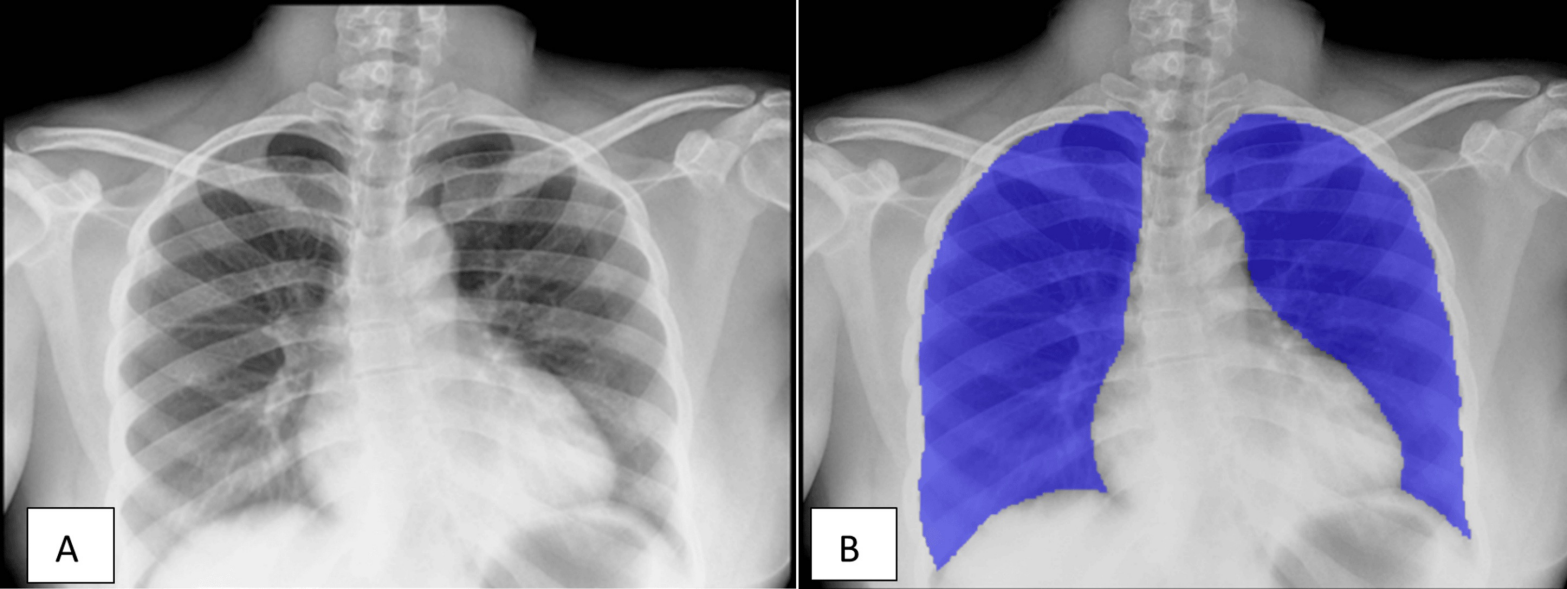
Case 2 chest X-ray: pulmonologist vs. CAD interpretation (A) Pulmonologist chest X-ray interpretation; normal chest X-ray, no abnormalities noted. (B) Chest X-ray with a CAD heat map; blue color indicating normal lung parenchyma.

On chest X-ray examination, both the pulmonologist's interpretation and the interpretation with CAD reviewed a normal X-ray (Figure 2). The patient was started on a fixed-dose combination of anti-TB treatment for drug-sensitive TB containing rifampicin 600 mg, pyrazinamide 1600 mg, ethambutol 1100 mg, and isoniazid 300 mg per day based on weight at the time of diagnosis.

The patient completed their treatment after six months and was cured at the end of the treatment duration, with sputum microscopy smear negative at the end of treatment. A contact investigation was conducted on household contacts, but did not yield any positive cases.

## Discussion

Sub-clinical TB poses a unique challenge due to its complex diagnosis and its potential role in TB transmission and progression to active disease [9]. These case reports underscore the challenge in identifying these asymptomatic TB patients and the importance of targeted screening strategies for high-risk individuals. Furthermore, it emphasizes the additive value of using chest X-ray with CAD. They further highlight the role of contact tracing, early treatment of asymptomatic TB cases, and the importance of adherence counselling and tools to improve treatment completion in this population. The two case studies have demonstrated complexity in identifying subclinical cases, with both cases presenting no recognizable symptoms and one case remaining asymptomatic for a prolonged period from initial diagnosis. This is in line with existing literature, which indicates that the transition from subclinical to clinical TB involves complex immunological changes that are not yet fully understood [10]. Some studies have postulated that this may be due to the imbalance between the host immune response and bacterial activity, and as the immune system weakens or the bacterial load increases, the disease progresses to an active, symptomatic state [3,11-13]. However, not all subclinical TB cases will progress to active TB. Overall, it is approximated that only 25-30% of individuals with subclinical TB in the general population will progress to active TB, while there is a 10% annual risk of progression in HIV-positive individuals [3,14].

To break the chain of transmission of TB, these asymptomatic patients will need to be identified and started on treatment. Most recently, the WHO has further classified asymptomatic TB into asymptomatic bacteriologically confirmed (positive rapid tests, e.g., Xpert or on culture or smear) and asymptomatic bacteriologically unconfirmed (radiological features suggestive of TB without bacteriological confirmation), in which the former should be managed with full active TB regimes while the later be worked up to rule out other alternative diagnoses such as silicosis, blastomycosis.

However, the specific diagnostic tools for the screening and diagnosis of subclinical TB are not readily available.

The role of chest X-rays, particularly when combined with CAD, plays a crucial role in the routine screening of high-risk individuals for TB [15]. CAD enhances the accuracy and efficiency of radiographic interpretation, making it easier to detect subtle abnormalities that may be missed by human readers and may indicate subclinical TB [8,16,17]. Routine screening of high-risk individuals (PLHIV, individuals living in TB-endemic areas, and healthcare workers) using chest X-rays with CAD can help identify TB cases that might otherwise go undetected on plain chest X-ray [17]. By incorporating CAD into routine screening protocols, healthcare systems can improve early detection rates and reduce the transmission of TB.

The potential for transmission from subclinical TB patients is a significant concern [1,2]. Contact tracing in this study yielded the diagnosis in two children, who were close contacts of one of the diagnosed individuals. This finding underscores the possibility of transmission from asymptomatic patients and highlights the importance of thorough contact investigations [1,18].

With the lack of symptoms seen in subclinical cases, there is a lower perception of the need for treatment, which can reduce motivation to adhere to lengthy and rigorous TB medication schedules [19]. In addition, the absence of immediate health deterioration might result in patients discontinuing treatment prematurely. This could potentially lead to an increased risk of drug-resistant TB. However, a study in rural South Africa showed a high treatment acceptance rate among asymptomatic microbiologically confirmed cases, although with unclear care pathways and linkage to care for this group. As subclinical cases are increasingly being identified, a lot of thought will need to go into building enough support systems to ensure adherence to treatment and defining clear linkage and care pathways.

Despite being able to identify these two cases, limitations should be acknowledged. Due to limited individual and system-level capacity, we were unable to subject the two cases to all possible diagnostic tools in identifying the other extrapulmonary lesions that could have been present; for example, CT scan and/or MRI for identifying spinal, abdominal, or adrenal TB; adrenal insufficiency testing, which could have pointed to the presence of adrenal insufficiency that was probably responsible for the change in skin complexion, vitamin D levels, and cytokines and other immunological markers, as these tests are generally not available and the cost are borne out of pocket by the patients who, majority of the time, are economically challenged.

## Conclusions

Subclinical TB represents a complex and underrecognized aspect of TB epidemiology. This case report highlights the clinical and probable epidemiological characteristics of subclinical TB cases, emphasizing the importance of early detection and intervention to reduce transmission and prevent the progression to active TB disease. TB programs in high-burden settings should consider screening among healthy high-risk individuals to identify and treat such individuals to reduce the risk of transmission and improve TB outcomes. As this is a case report, care must be taken not to generalize the findings; however, further research is needed to better understand the dynamics of subclinical TB, its immunology, and implications for TB control strategies.
